# Myocardial strain assessed by feature tracking cardiac magnetic resonance in patients with a variety of cardiovascular diseases – A comparison with echocardiography

**DOI:** 10.1038/s41598-019-47775-4

**Published:** 2019-08-05

**Authors:** Kasper Pryds, Anders Hostrup Larsen, Mona Sahlholdt Hansen, Anne Yoon Krogh Grøndal, Rasmus Stilling Tougaard, Nils Henrik Hansson, Tor Skibsted Clemmensen, Brian Bridal Løgstrup, Henrik Wiggers, Won Yong Kim, Hans Erik Bøtker, Roni Ranghøj Nielsen

**Affiliations:** 1 Department of Cardiology, Aarhus University Hospital, Aarhus, Denmark; 2 Department of Clinical Medicine, Aarhus University, Aarhus, Denmark; 3 Department of Medicine, Randers Regional Hospital, Randers, Denmark; 4 Department of Otorhinolaryngology, Aalborg University Hospital, Aalborg, Denmark

**Keywords:** Valvular disease, Heart failure

## Abstract

Myocardial deformation assessed by speckle tracking echocardiography (STE) is increasingly used for diagnosis, monitoring and prognosis in patients with clinical and pre-clinical cardiovascular diseases. Feature tracking cardiac magnetic resonance (FT-CMR) also allows myocardial deformation analysis. To clarify whether the two modalities can be used interchangeably, we compared myocardial deformation analysis by FT-CMR with STE in patients with a variety of cardiovascular diseases and healthy subjects. We included 40 patients and 10 healthy subjects undergoing cardiac magnetic resonance and echocardiographic examination for left ventricular volumetric assessment. We studied patients with heart failure and reduced ejection fraction (n = 10), acute perimyocarditis (n = 10), aortic valve stenosis (n = 10), and previous heart transplantation (n = 10) by global longitudinal (GLS), radial (GRS) and circumferential strain (GCS). Myocardial deformation analysis by FT-CMR was feasible in all but one participant. While GLS, GRS and GCS measured by FT-CMR correlated overall with STE (r = 0.74 and p < 0.001, r = 0.58 and p < 0.001, and r = 0.76 and p < 0.001), the correlations were not consistent within subgroups. GLS was systematically lower, whereas GRS and GCS were higher by FT-CMR compared to STE (p = 0.04 and p < 0.0001). Inter- and intra-observer reproducibility were comparable for FT-CMR and STE overall and across subgroups. In conclusion, myocardial deformation can be evaluated using FT-CMR applied to routine cine-CMR images in patients with a variety of cardiovascular diseases. However, correlation between FT-CMR and STE was modest and agreement was not optimal due to systematic bias regarding GLS and GCS. Consequently, FT-CMR and STE should not be used interchangeably for myocardial strain evaluation.

## Introduction

Myocardial deformation analysis enables quantification of left ventricular (LV) contractile function and has been proven to enhance risk stratification of patients with overt cardiovascular disease as well as improve the identification of subclinical LV systolic dysfunction compared to conventional echocardiographic parameters^1,2^. Speckle tracking echocardiography (STE) is routinely used for myocardial deformation analysis in clinical practice. STE is an easily accessible and low-cost procedure with no patient discomfort. Feature tracking cardiac magnetic resonance (FT-CMR) can be applied to routine cine-cardiac magnetic resonance (CMR) acquisitions^3^ and enables myocardial deformation analysis without extending the image protocol or the duration of the CMR examination. Both STE and FT-CMR allows for offline assessment of myocardial deformation, resulting in easier access and wider availability^4^. Myocardial deformation analysis by STE may be compromised by suboptimal image quality due to insufficient acoustic windows, ultrasound dropouts or reverberations^4^. In patients with suboptimal image quality, FT-CMR may improve myocardial deformation analysis compared to STE, rendering FT-CMR an attractive tool for assessment of myocardial strain for clinical and research purposes. However, comparison of myocardial deformation analysis using FT-CMR with STE in different patient groups is sparse. The aim of the present study was to compare diagnostic accuracy and inter-and intra-observer reproducibility between post-processing myocardial deformation analysis by FT-CMR and STE in patients with a variety of cardiovascular diseases and in healthy subjects.

## Methods

### Design and study population

We included 50 participants previously enrolled as part of clinical trials conducted at Department of Cardiology, Aarhus University Hospital, Denmark: 10 patients with compensated heart failure (HF)^5^, 10 with acute perimyocarditis^6^, 10 with aortic valve stenosis (AVS)^7^, 10 with heart transplantation (HTX) and 10 healthy subjects^7^. All participants underwent CMR examination for evaluation of LV volumetric parameters, whereas echocardiographic examination was conducted for evaluation of both LV volumetric as well as myocardial deformation parameters.

All participants underwent both CMR and echocardiography examinations within a mean period of 3 ± 10 days for evaluation of intra- and inter-observer reproducibility for myocardial deformation analysis. In addition, for evaluation of test-retest reliability, healthy subjects underwent CMR and echocardiographic examinations on two separate occasions within a mean period of 6 ± 1 days. For evaluation of intra- and inter-observer reproducibility, we aimed to reflect normal clinical practice. Consequently, the choice of CMR frame and echocardiography image for myocardial deformation analysis was at the discretion of the observer. The observers (FT-CMR; MSH and AYKG, and STE; AHL and RST) were blinded to all clinical and previous imaging data and not involved in any imaging acquisition.

### Feature tracking cardiac magnetic resonance imaging

All CMR examinations were performed on a Philips Achieva dStream 1.5 T scanner system (Philips Healthcare, Best, The Netherlands) with a 60 cm bore. Anterior and posterior coil arrays were used. Cine-CMR examinations were conducted electrocardiogram-triggered and performed during breath-hold. Thirty phases were derived for each cardiac cycle. We used the commercially available software system Segment version 2.0 (Medviso AB, Lund, Sweden) for FT-CMR data analysis^8^.

Offline FT-CMR analyses were conducted for evaluation of peak global longitudinal strain (GLS), global radial strain (GRS) and global circumferential strain (GCS) in a 17-segment software-generated model. For GLS, data on myocardial strain were derived from two-, three- and four-chamber long-axis views. However, three-chamber long-axis views were not available among patients with heart failure, in whom GLS was derived from two- and four-chamber long-axis views only. For GRS and GCS, data on myocardial strain were derived from apical, mid-ventricular and basal short-axis views in all patients. On all images, the epi- and endocardial borders were outlined in the end-diastole. Subsequently, an automatic computation was triggered, by which the applied software algorithm automatically outlined the border throughout the cardiac cycle. The quality of the tracking and contouring was visually validated and manually corrected if needed. Additional cine-CMR data analyses were done for evaluation of left ventricular ejection fraction (LVEF) as previously described^9^.

### Speckle tracking echocardiography

All transthoracic echocardiographic examinations were performed on a GE Vivid E9 ultrasound system (GE Healthcare, Chicago, Illinois, USA) with a 2.5 MHz transducer and image acquisition according to the current international guidelines^10^. We used the commercially available software system EchoPAC version 202, revision 34.0 (GE Healthcare, Chicago, Illinois, USA) for STE data analysis.

Offline two-dimensional high-resolution STE (>50 frames per second) analyses were conducted for evaluation of peak systolic GLS based on a 17-segment software-generated model of the left ventricle derived from apical two-, three- and four-chamber views. GRS and GCS were calculated as average strain values based on apical, mid-ventricular and basal short-axis parasternal views. The region of interest (ROI) included the complete left-side myocardium and myocardial strain was calculated as an average of the mid myocardial layers. Prior to myocardial tracking, aortic valve opening and closure were defined by the event timing function. Subsequent calculation of peak systolic myocardial strain was software-generated using the automated function Q-Analysis module^11^.

### Statistics

Unpaired data were tested for normal distribution and equality of variance, and paired data were tested for normal distribution and equality and normality of variance prior to statistical analysis. Categorical variables were compared using χ^2^ test or Fisher’s exact test. Paired continuous variables were analysed using paired Student’s t-test. Unpaired continuously variables were analysed using unpaired Student’s t-test, one-way analysis of variance (ANOVA) or Kruskal Wallis test in case of unequal variance.

Agreement between FT-CMR and STE myocardial strain evaluation was assessed using linear regression analysis and Bland-Altman plots. Paired Student’s t-test was used to evaluate the presence of systematic differences between FT-CMR and STE^12^. Inter-observer (two readers) and intra-observer (two readings) reproducibility and test-retest reliability were assessed using the standard error of estimate, coefficient of variation (CV) and intraclass correlation coefficient (ICC).

Statistical analyses were conducted using STATA/SE 15 (StataCorp, College Station, USA). Data are presented as numbers (%) or mean ± standard deviation and with 95% confidence interval (CI) when appropriate. Statistical significance was set as two-sided p-value of < 0.05.

### Ethics

Data were collected according to the study protocols at the Department of Cardiology, Aarhus University Hospital, Denmark. All CMR and STE examinations were carried out in accordance with relevant guidelines and regulations. The studies were carried out in accordance with the Declaration of Helsinki (2008) of the World Medical Association, and were approved by The Central Denmark Region Committees on Health Research Ethics and The Danish Data Protection Agency. All participants gave written informed consent prior to inclusion.

## Results

**Figure 1:**
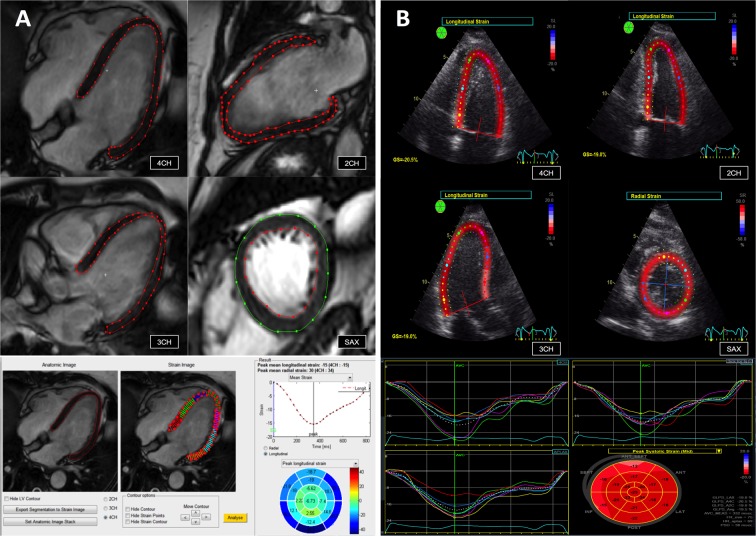
Myocardial strain assessment by FT-CMR and STE. Illustration depicting myocardial segmentation and data analysis for global longitudinal strain evaluation using feature tracking cardiac magnetic resonance imaging (**A**) and 2-D speckle tracking echocardiography (**B**). See text for details. 4CH, Four-chamber view; 2CH, Two-chamber view; 3CH, Three-chamber view; SAX, Short-axis view.

Descriptive baseline characteristics for the 50 participants included in the study are listed in Table 1. Offline myocardial deformation evaluation was successfully calculated using FT-CMR in all participants except for GLS in a single participant for assessment of inter-observer reproducibility. STE-derived GLS was feasible in all patients. Due to insufficient echocardiographic image quality and consequent compromised myocardium tracking, STE-derived GRS and GCS were obtained in only 39 (78%) participants for intra-observer reproducibility analysis and in 33 (66%) participants for inter-observer reproducibility analysis (Fig. 1).

**Table 1:** Baseline characteristics.

	Healthy subjects	HF	Peri-myocarditis	AVS	HTX	p-value
	(n = 10)	(n = 10)	(n = 10)	(n = 10)	(n = 10)	
Age (years)	63.1 ± 4.4	67.9 ± 5.0	29.7 ± 12.6	71.1 ± 8.7	54.0 ± 10.3	0.0001
Men	7 (70%)	10 (100%)	6 (60%)	5 (50%)	8 (80%)	0.11
Body mass index (kg/meter^2^)	25.9 ± 4.0	28.9 ± 5.1	24.8 ± 2.4	25.3 ± 4.3	26.2 ± 3.9	0.20
Comorbidity:						
Hypertension	0 (0%)	3 (30%)	0 (0%)	4 (40%)	8 (80%)	<0.001
Atrial fibrillation	0 (0%)	0 (0%)	0 (0%)	0 (0%)	0 (0%)	—
Previous acute myocardial infarction	0 (0%)	8 (80%)	0 (0%)	0 (0%)	0 (0%)	<0.001
Medical treatment:						
Beta-blockers	0 (0%)	10 (100%)	0 (0%)	0 (0%)	0 (0%)	<0.001
ACE-I/ARB	0 (0%)	10 (100%)	0 (0%)	0 (0%)	4 (40%)	<0.001
Cardiac device therapy*	0 (0%)	0 (0%)	0 (0%)	0 (0%)	0 (0%)	—
Biochemical and hemodynamic parameters:						
eGFR >90 (ml/min/1.73 m^2^)	10 (100%)	5 (50%)	6 (60%)	5 (50%)	2 (20%)	0.005
LVEF (%)	65.9 ± 3.3	42.2 ± 5.8	56.9 ± 14.4	66.6 ± 19.7	66.4 ± 14.8	0.001

Data are mean ± SD or absolute numbers (%). HF, heart failure; AVS, Aortic valve stenosis; HTX, heart transplant; ACE-I, Angiotensin-converting enzyme inhibitor; ARB, Angiotensin II receptor blockers; eGFR, estimated glomerular filtration rate; LVEF, left ventricular ejection fraction.

*Bradypacemaker, Cardiac resynchronization therapy or implantable cardiac defibrillator.

### Myocardial strain evaluation using FT-CMR compared to STE

**Figure 2:**
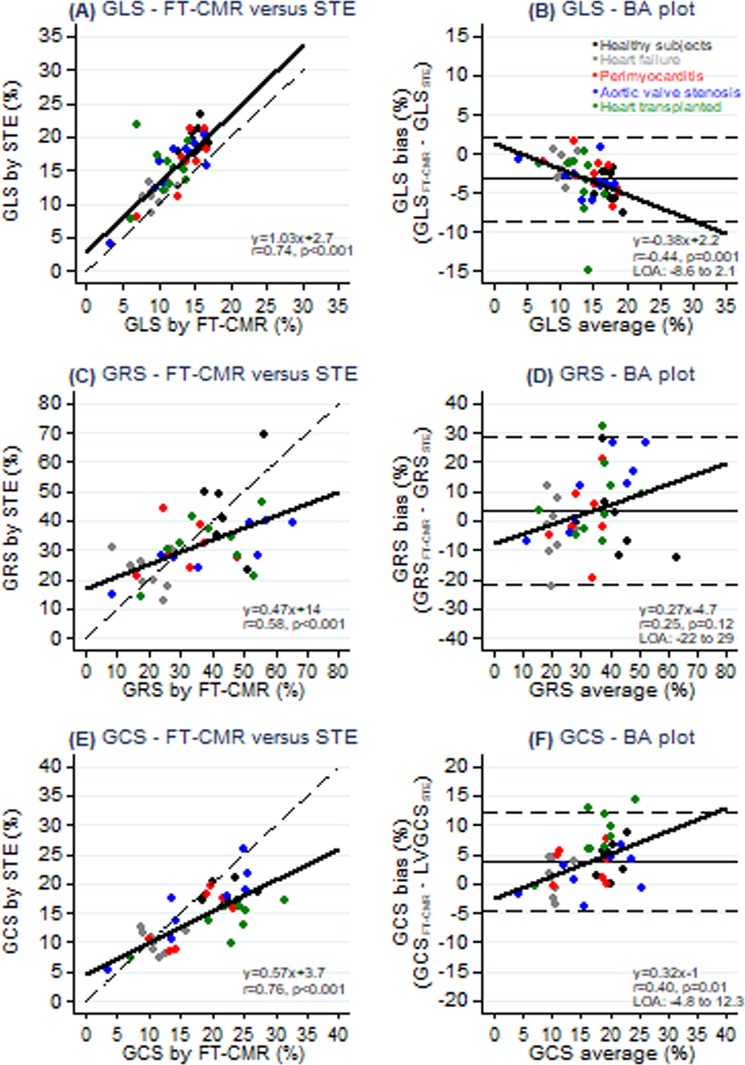
Myocardial strain evaluation using FT-CMR vs. STE. FT-CMR, Feature tracking cardiac magnetic resonance; STE, speckle tracking echocardiography; GLS, Global longitudinal strain; GRS, Global radial strain; GCS, Global circumferential strain; BA, Bland-Altman; LOA, 95% limits of agreements (mean ± 1.96*standard deviation).

Overall, GLS, GRS and GCS measured by FT-CMR correlated with STE (r = 0.74, p < 0.001, r = 0.58, p < 0.001, and r = 0.76, p < 0.001) (Fig. 2 and Table 2). GLS was systematically lower (12.5 ± 3.1 vs. 15.6 ± 4.3; 95% CI −3.9 to −2.3, p < 0.0001), whereas GRS (34.8 ± 14.5 vs. 30.6 ± 12.0; 95% CI 0.2 to 8.2, p = 0.04) and GCS (18.3 ± 6.4 vs. 14.2 ± 4.8; 95% CI 2.8 to 5.5, p < 0.0001) were higher by FT-CMR than by STE (Table 2). In addition, GLS and GCS quantification revealed proportional bias (Fig. 2) and demonstrated that limits of agreement were lowest for GLS and GCS.

**Table 2:** Myocardial strain evaluation using FT-CMR vs. STE.

	Difference				
	FT-CMR	STE	Absolute	95% CI	*p*
All participants					
GLS (n = 50)	12.5 ± 3.1	15.6 ± 4.3	−3.1 ± 2.9	−3.9 to −2.3	<0.0001
GRS (n = 39)	34.8 ± 14.5	30.6 ± 12.0	4.2 ± 12.3	0.2 to 8.2	0.04
GCS (n = 39)	18.3 ± 6.4	14.2 ± 4.8	4.1 ± 4.2	2.8 to 5.5	<0.0001
	**Correlation**				
	**Slope**	**SEE**	**Intercept**	***r***	***p***
GLS (n = 50)	1.0	2.9	2.7	0.74	<0.001
GRS (n = 39)	0.5	9.7	14.2	0.58	<0.001
GCS (n = 39)	0.6	3.2	3.8	0.76	<0.001

Data are mean ± SD or absolute numbers.

FT-CMR, Feature tracking cardiac magnetic resonance; STE, speckle tracking echocardiography; CI, confidence interval; SEE, Standard error of estimate; GLS, Global longitudinal strain; GRS, Global radial strain; GCS, Global circumferential strain.

Subgroup analyses showed that correlations between FT-CMR and STE were statistically significant for GLS among patients with perimyocarditis and AVS, among patients with AVS for GRS, and among patients with perimyocarditis, AVS and HTX for GCS. Correlations were not statistically significant in the remaining subgroups. Results among participant subgroups are shown in Supplementary Tables 1–3.

### Intra- and inter-observer reproducibility for myocardial strain evaluation using FT-CMR and STE

Data on FT-CMR- and STE-derived reproducibility are listed in Table 3. Overall, intra- and inter-observer reproducibility for GLS, GRS and GLS were comparable by FT-CMR and STE. FT-CMR and STE intra-observer reproducibility was excellent for GLS, GRS and GCS, whereas FT-CMR and STE inter-observer reproducibility was excellent for GLS and GCS and good for GRS. Results among participant subgroups are shown in Supplementary Tables 4–5.

**Table 3:** Intra-observer and inter-observer reproducibility for myocardial strain evaluation using FT-CMR and STE.

	Intra-observer			Inter-observer		
	SEE	CV (%)	ICC	SEE	CV (%)	ICC
**FT-CMR**						
Overall						
GLS	0.5 (n = 50)	4.2 (n = 50)	0.99 (n = 50)	1.2 (n = 49)	9.1 (n = 49)	0.95 (n = 49)
GRS	4.5 (n = 50)	12.8 (n = 50)	0.98 (n = 50)	4.7 (n = 50)	20.2 (n = 50)	0.84 (n = 50)
GCS	1.3 (n = 50)	6.8 (n = 50)	0.99 (n = 50)	2.2 (n = 50)	13.1 (n = 50)	0.94 (n = 50)
**STE**						
Overall						
GLS	0.6 (n = 50)	4.2 (n = 50)	0.99 (n = 50)	1.5 (n = 50)	9.5 (n = 50)	0.97 (n = 50)
GRS	4.2 (n = 39)	15.1 (n = 39)	0.96 (n = 39)	8.1 (n = 33)	26.7 (n = 33)	0.88 (n = 33)
GCS	0.9 (n = 39)	6.3 (n = 39)	0.99 (n = 39)	2.2 (n = 33)	16.9 (n = 33)	0.93 (n = 33)

Data are absolute numbers.

FT-CMR, Feature tracking cardiac magnetic resonance; STE, speckle tracking echocardiography; SEE, Standard error of estimate; CV, coefficient of variation; ICC, Intraclass correlation coefficient; GLS, Global longitudinal strain; GRS, Global radial strain; GCS, Global circumferential strain.

### Test-retest reliability for myocardial strain evaluation using FT-CMR and STE

FT-CMR-derived test-retest data on GLS, GRS and GCS were obtained for all 10 healthy subjects, whereas STE-derived GLS, GRS and GCS data were obtained for 10 (100%), 5 (50%) and 5 (50%) healthy subjects, respectively. Test-retest reliability was good for FT-CMR-derived GLS (ICC: 0.88) and STE-derived GLS (ICC: 0.87) and GRS (ICC: 0.88). In contrast, FT-CMR-derived GRS (ICC: 0.16) and GCS (ICC: 0.44) and STE-derived GCS (ICC: 0.38) were poor (Supplementary Table 6).

## Discussion

In the present study, we evaluated the performance of offline FT-CMR analysis for myocardial deformation assessment and compared it to STE in patients with a variety of cardiovascular diseases and in healthy subjects. Importantly, the echocardiographic examinations were specifically performed to assess strain, whereas CMR was performed to assess volumetric measurements. While strain measurements derived from FT-CMR and STE correlated overall, the correlations were not consistent among all subgroups and agreement was not optimal due to systematic bias regarding GLS and GCS. FT-CMR and STE intra- and inter-observer reproducibility were excellent for GLS and GCS. Test-retest reliability was good by FT-CMR- and STE-derived GLS, whereas test-retest reliabilities for FT-CMR-derived GRS and GCS were poor.

### Intermodality agreement between FT-CMR and STE for myocardial deformation analysis

Myocardial strain is a more sensitive marker of LV dysfunction than LVEF^13^. Intermodality agreement between FT-CMR and STE is superior for GLS than between cine-CMR and 2-dimensional echocardiography for LVEF^14^. Thus, in clinical practice, myocardial deformation analysis can be used in the diagnosis of patients with sub-clinical LV dysfunction^15^. Myocardial strain analysis has proved to be an important prognostic marker of mortality and adverse cardiovascular events superior to LVEF in various cardiovascular diseases, including acute coronary syndrome, HF, valvular heart disease^1^ and HTX^16^. Furthermore, myocardial strain evaluation is a promising tool to diagnose myocardial ischemia and guide cardiac lead placement in resynchronization therapy^15^. Compared to echocardiography, CMR for myocardial deformation analysis is (I) time consuming, (II) challenging or impossible in patients with claustrophobia or implanted metal devices^17^, and (III) limited by a lack of validation and general availability. Consequently, STE has been the primary tool used for myocardial deformation analysis. However, in both clinical practice and for research purposes, CMR offers several advantages compared to echocardiography, including superior myocardial volumetric and functional assessment, while at the same time enabling evaluation of myocardial perfusion and tissue composition. In the present study, the cine-CMR examination was intended for LV volumetric assessment. Despite this, off-line FT-CMR assessment of GLS, GRS and GCS was feasible in almost all cases irrespective of the specific underlying cardiovascular disease. Although STE examinations were conducted for evaluation of myocardial deformation parameters, STE requires optimal image acquisition for myocardial deformation analysis, which compromised retrospective strain assessment in a considerable proportion of the included participants.

Both FT-CMR and STE rely on tissue-tracking image post-processing methods based on patterns in the myocardium being tracked during the cardiac cycle^18^. While STE relies on real-time images, FT-CMR relies on cine-CMR images from different cardiac cycles. STE software offers automatic detection of the myocardium and definition of aortic valve opening and closure. In contrast, FT-CMR requires manual tracing of the endo- and epicardial borders and definition of end-diastole based on visual assessment of LV dimensions^14^. Validation of FT-CMR compared to STE has previously been conducted in patients with hypertrophic cardiomyopathy^19^, constrictive pericarditis^20^, restrictive cardiomyopathy^20^, repaired tetralogy of Fallot^21,22^, in unselected cohorts of patients with a variety of cardiovascular diseases, including heart failure^14,23^, and in healthy subjects^19–22^. We extended previous findings by comparing myocardial strain assessment by FT-CMR with STE in patients with perimyocarditis, aortic valve stenosis and HTX. The lack of significant correlations between FT-CMR and STE strain values within subgroups may not only be due to limited sample size and considerable variability of the measurements but also due to the systematic bias that we detected for agreement between the two modalities. However, due to the limited number of participants, the subgroup analyses should be interpreted with caution.

The results of the present study demonstrate that offline FT-CMR can be applied to routine cine-CMR acquisitions for evaluation of myocardial deformation analysis in patients with a variety of cardiovascular diseases. Consequently, myocardial deformation assessment by FT-CMR is feasible without extending the magnetic resonance  acquisition protocol. As with STE, FT-CMR may be used for single measurements as well as for serial measurements of myocardial strain. However, because FT-CMR underestimates GLS while overestimating GRS and GCS compared to STE, FT-CMR and STE should not be used interchangeably for myocardial strain evaluation.

### Reproducibility of myocardial deformation analysis using FT-CMR

We demonstrated that GLS has the best intra- and inter-observer reproducibility followed by GCS and GRS by both FT-CMR and STE. Our results are in accordance with previously reported FT-CMR intra-observer reproducibility^4^. Overall, we found intra- and inter-observer CV of 4.2% and 9.1% for GLS, 12.8% and 20.2% for GRS, and 6.8% and 13.1% for GCS using FT-CMR while similar numbers were 4.2% and 9.5%, 15.1% and 26.7%, and 6.3% and 16.9% using STE. In comparison, LVEF intra- and inter-observer CV has been found to be 1.3% and 4.3% using cine-CMR and 6.9% and 8.1% using 2-dimensional echocardiography in an unselected cohort of patients with cardiovascular diseases^14^. In another unselected cohort of patients with a variety of cardiovascular diseases and healthy volunteers, LVEF intra- and inter-observer CV was found to be 5.1% and 3.6% using cine-CMR, 13.4% and 17.8% using 2-dimensional echocardiography, and 6.9% and 8.3% using 3-dimensional echocardiography^24^. In another study on unselected patients with cardiovascular diseases, LVEF intra- and inter-observer CV was  9.0% and 12.0% by 2-dimensional echocardiography and 5.3% and 9.3% using 3-dimensional echocardiography^25^. Furthermore, we extended previous findings by comparing test-retest reliability by FT-CMR and STE in healthy subjects. We demonstrated that GLS has acceptable and comparable test-retest reliability by FT-CMR and STE, whereas test-retest reliability for GCS was poor by both FT-CMR and STE. In the present study, we found test-retest CV of 5.6%, 28.5% and 14.6% for GLS, GRS and GCS respectively using FT-CMR while similar numbers were 5.0%, 21.9% and 16.7% using STE. In comparison, LVEF test-retest CV has been found to be 2.4% and 8.6% using cine-CMR and 2-dimensional echocardiography, respectively^26^. Compared to previous studies, FT-CMR and STE CV were overall higher in the present study, in particular inter-observer CV for GRS and GCS. This may likely to be due to the fact that the choice of CMR frame and echocardiography image for myocardial deformation analysis was at the discretion of the observer in the present study. This approach reflects normal clinical practice. Therefore, our findings question the ability to detect minor serial changes in GRS and GCS using FT-CMR with the current software implementation and standard cine-CMR protocol. Use of a cine-CMR imaging protocol with an increased number of cardiac phases per cardiac cycle may improve myocardial deformation assessment by FT-CMR.

The present study demonstrates that while myocardial strain evaluation is feasible by offline FT-CMR analysis using conventional cine-CMR examinations conducted for assessment of volumetric parameters, FT-CMR is not superior regarding either intra-observer or inter-observer reproducibility or test-retest reliability compared to STE. This finding is in contrast to previous findings by Onishi and colleagues, who demonstrated that reproducibility was slightly better by FT-CMR in an unselected cohort of patients with a variety of cardiovascular diseases^23^. Importantly, while FT-CMR offers superior signal to noise ratio, FT-CMR yields lower in-plane spatial and temporal resolution compared to STE^4^. In addition, FT-CMR may be compromised within the compact myocardium of the left ventricle^18^. These limitations of FT-CMR may explain our observation that inter- and intra-observer as well as test-retest reliability is non-superior by FT-CMR compared to STE. As demonstrated in the present study, STE for myocardial deformation assessment may be compromised due to insufficient echocardiographic image quality especially for GRS and GCS. In contrast, FT-CMR-derived myocardial deformation analysis was feasible in all except one participant. Consequently, myocardial strain evaluation by FT-CMR may be superior in case of suboptimal image quality for STE. Future studies should identify specific patients characteristics for which FT-CMR may improve myocardial deformation analysis compared to STE, e.g. patients with myocardial deposition, obesity or pulmonary disease. Strain-encoded magnetic resonance (SENC) is a promising method for detection and risk stratification of patients with a variety of cardiovascular diseases^27^. SENC is an advanced myocardial tagging technique for comprehensive myocardial deformation analysis and holds important advantages compared to other methods for myocardial strain evaluation, including real-time strain assessment and single-heartbeat and free breathing acquisitions^27,28^. Furthermore, SENC benefits from short acquisition and post-processing times^28^, and is highly reproducible^29^. However, clinical validation of SENC is sparse.

### Limitations

Important limitations of the present study include the retrospective design, and the fact that we compared myocardial deformation analysis by FT-CMR with STE and not CMR tagging method, which is considered the gold standard for assessment of myocardial strain^18^. However, CMR tagging is time consuming and requires dedicated image acquisition^13^. Thus, STE is the predominant imaging modality used for myocardial deformation analysis in clinical practice. Further validation and improvement of myocardial deformation analysis using FT-CMR is needed to facilitate widespread application in clinical practice and for research purposes.

In the present study, two vendors for analysis of myocardial strain were used, i.e. EchoPAC for STE and Segment for FT-CMR. Each vendor utilizes different algorithms for myocardial deformation analysis. This may have impacted our results by inherently increasing the differences between the present STE and FT-CMR measurements.

The study carries risk of selection bias as only patients suitable for CMR examinations were included in the parent trials, and patients with insufficient acoustic windows were *per se* excluded. Consequently, FT-CMR was compared to a setting of optimal STE conditions. In addition, because strain values differed between participant subgroups, the overall correlation analyses should be interpreted with caution. Finally, test-retest reliability analysis was only available for healthy subjects and future studies should assess test-retest reliability in patients with cardiovascular diseases.

## Conclusion

Our study demonstrates that myocardial deformation can be evaluated using FT-CMR applied to routine cine-CMR images in patients with a variety of cardiovascular diseases. Correlation between FT-CMR and STE was modest and agreement was not optimal due to systematic bias regarding GLS and GCS. Consequently, FT-CMR and STE should not be used interchangeably for myocardial strain evaluation. However, FT-CMR and STE had comparable inter- and intra-observer reproducibility, and GLS had good test-retest reliability by FT-CMR and STE, whereas test-retest reliability for FT-CMR-derived GRS and GCS were poor.

## Supplementary information

Supplementary Dataset 1

## Data Availability

The datasets used and/or analysed during the current study are available from the corresponding author on reasonable request.
