# Supplementary material for: Myocardial strain assessed by feature tracking cardiac magnetic resonance in patients with a variety of cardiovascular diseases – A comparison with echocardiography
Source: Sci Rep. 2019 Aug 5;9:11296. doi: 10.1038/s41598-019-47775-4 (PMC6683180; doi:10.1038/s41598-019-47775-4)
Supplement: Supplementary file 1 — Supplementary Dataset 1 [file 41598_2019_47775_MOESM1_ESM.pdf]

Myocardial strain assessed by feature tracking cardiac magnetic resonance in patients with a variety of cardiovascular diseases – A comparison with echocardiography

Kasper Pryds, MD, PhD[1,2,3], Anders Hostrup Larsen, MD[1,2], Mona Sahlholdt Hansen, MD[1,2], Anne Yoon Krogh Grøndal[4], MD, Rasmus Stilling Tougaard[1,2], MD, PhD, Nils Henrik Hansson, MD, PhD[1], Tor Skibsted Clemmensen, MD, PhD[1], Brian Bridal Løgstrup, MD, PhD, DMSc[1], Professor Henrik Wiggers, MD, PhD, DMSc[1], Professor Won Yong Kim, MD, PhD, DMSc[1], Professor Hans Erik Bøtker, MD, PhD, DMSc[1], Roni Ranghøj Nielsen, MD, PhD[1,2]

[1]Department of Cardiology, Aarhus University Hospital, Aarhus, Denmark, [2]Department of Clinical Medicine, Aarhus University, Aarhus, Denmark, [3]Department of Medicine, Randers Regional Hospital, Randers, Denmark & [4]Department of Otorhinolaryngology, Aalborg University Hospital, Aalborg, Denmark

Correspondence to:

Kasper Pryds, MD, PhD, Department of Cardiology, Aarhus University Hospital, Palle Juul-Jensens Boulevard 99, DK-8200 Aarhus N, Denmark; Phone: +4575482029; E-mail: kpryds@clin.au.dk

Supplementary table 1 – Myocardial global longitudinal strain evaluation using FT-CMR vs. STE among participant subgroups

	**Difference**				
	**FT-CMR**	**STE**	**Absolute**	**95% CI**	***p***
**GLS**					
– Overall (n=50)	12.5 ± 3.1	15.6 ± 4.3	-3.1 ± 2.9	-3.9 to -2.3	<0.0001
– Healthy subjects (n=10)	15.1 ± 1.2	19.4 ± 1.9	-4.3 ± 1.9	-5.6 to -2.9	0.001
– HF (n=10)	10.4 ± 1.4	11.4 ± 1.6	-1.0 ± 2.0	-2.4 to 0.4	0.14
– Perimyocarditis (n=10)	13.7 ± 3.0	16.5 ± 4.4	-2.8 ± 2.4	-4.5 to -1.1	0.005
– AVS (n=10)	12.2 ± 4.0	15.5 ± 4.7	-3.3 ± 2.1	-4.8 to -1.8	0.001
– HTX (n=10)	11.0 ± 2.7	15.1 ± 4.0	-4.1 ± 4.5	-7.2 to -0.9	0.02
	**Correlation**				
	**Slope**	**SEE**	**Intercept**	***r***	***p***
– Overall (n=50)	1.0	2.9	2.7	0.74	<0.001
– Healthy subjects (n=10)	0.5	1.9	12.1	0.32	0.38
– HF (n=10)	0.2	1.7	9.6	0.16	0.66
– Perimyocarditis (n=10)	1.3	2.3	-1.1	0.87	0.001
– AVS (n=10)	1.1	2.2	2.6	0.89	<0.001
– HTX (n=10)	0.2	4.2	12.6	0.15	0.67

Data are mean ± SD or absolute numbers.

FT-CMR, Feature tracking cardiac magnetic resonance; STE, speckle tracking echocardiography; CI, confidence interval; SEE, Standard error of estimate; GLS, Global longitudinal strain; HF, heart failure; AVS, Aortic valve stenosis; HTX, heart transplantation.

Supplementary table 2 – Myocardial global radial strain evaluation using FT-CMR vs. STE among participant subgroups

			Difference		
	**CMR**	**STE**	**Absolute**	**95% CI**	***p***
**GRS**					
– Overall (n=39)	34.8 ± 14.5	30.6 ± 12.0	4.2 ± 12.3	0.2 to 8.2	0.04
– Healthy subjects (n=7)	42.8 ± 9.1	42.3 ± 15.4	0.54 ± 14.1	-12.5 to 13.6	0.92
– HF (n=8)	20.2 ± 4.8	19.3 ± 7.3	0.8 ± 8.3	-6.1 to 7.7	0.79
– Perimyocarditis (n=7)	31.7 ± 10.4	30.8 ± 8.2	1.0 ± 12.7	-10.8 to 12.7	0.85
– AVS (n=8)	40.6 ± 19.6	30.2 ± 8.7	10.5 ± 12.9	-0.3 to 21.3	0.06
– HTX (n=9)	38.8 ± 13.1	31.7 ± 9.8	7.0 ± 12.8	-2.8 to 16.8	0.14
			**Correlation**		
	**Slope**	**SEE**	**Intercept**	***r***	***p***
– Overall (n=39)	0.5	9.7	14.2	0.58	<0.001
– Healthy subjects (n=7)	0.7	15.2	11.2	0.43	0.34
– HF (n=8)	0.2	7.8	15.7	0.12	0.78
– Perimyocarditis (n=7)	0.1	8.9	28.8	0.08	0.87
– AVS (n=8)	0.4	4.8	14.7	0.86	0.01
– HTX (n=9)	0.3	9.6	19.9	0.41	0.28

Data are mean ± SD or absolute numbers.

FT-CMR, Feature tracking cardiac magnetic resonance; STE, speckle tracking echocardiography; CI, confidence interval; SEE, Standard error of estimate; GRS, Global radial strain; HF, heart failure; AVS, Aortic valve stenosis; HTX, heart transplantation.

Supplementary table 3 – Myocardial global circumferential strain evaluation using FT-CMR vs. STE among participant subgroups

			Difference		
	CMR	STE	Absolute	95% CI	*p*
**GCS**					
– Overall (n=39)	18.3 ± 6.4	14.2 ± 4.8	4.1 ± 4.2	2.8 to 5.5	<0.0001
– Healthy subjects (n=7)	22.5 ± 2.8	18.2 ± 1.9	4.2 ± 3.1	1.3 to 7.1	0.01
– HF (n=8)	12.1 ± 2.2	9.3 ± 1.9	2.8 ± 3.0	0.3 to 5.3	0.03
– Perimyocarditis (n=7)	17.3 ± 4.9	14.1 ± 4.7	3.2 ± 3.1	0.4 to 6.1	0.03
– AVS (n=8)	17.9 ± 7.9	16.4 ± 6.4	1.5 ± 3.6	-1.5 to 4.5	0.26
– HTX (n=9)	21.9 ± 6.6	13.6 ± 3.2	8.3 ± 4.5	4.8 to 11.7	0.001
			**Correlation**		
	**Slope**	**SEE**	**Intercept**	***r***	***p***
– Overall (n=39)	0.6	3.2	3.8	0.76	<0.001
– Healthy subjects (n=7)	0.1	2.0	15.7	0.17	0.72
– HF (n=8)	-0.1	2.0	10.2	-0.1	0.85
– Perimyocarditis (n=7)	0.8	3.1	0.8	0.80	0.03
– AVS (n=8)	0.7	3.1	3.3	0.90	0.003
– HTX (n=9)	0.4	2.1	5.3	0.79	0.01

Data are mean ± SD or absolute numbers.

FT-CMR, Feature tracking cardiac magnetic resonance; STE, speckle tracking echocardiography; CI, confidence interval; SEE, Standard error of estimate; GCS, Global circumferential strain; HF, heart failure; AVS, Aortic valve stenosis; HTX, heart transplantation.

Supplementary table 4 – Intra-observer and inter-observer reproducibility for myocardial strain evaluation using FT-CMR among participant subgroups

	Intra-observer			Inter-observer		
	SEE	CV (%)	ICC	SEE	CV (%)	ICC
**GLS**						
Overall	0.5 (n=50)	4.2 (n=50)	0.99 (n=50)	1.2 (n=49)	9.1 (n=49)	0.95 (n=49)
– Healthy subjects	0.6 (n=10)	3.7 (n=10)	0.95 (n=10)	0.6 (n=10)	3.5 (n=10)	0.83 (n=10)
– HF	0.3 (n=10)	2.8 (n=10)	0.99 (n=10)	0.7 (n=9)	6.2 (n=9)	0.96 (n=9)
– Perimyocarditis	0.5 (n=10)	5.1 (n=10)	0.99 (n=10)	0.9 (n=10)	4.8 (n=10)	0.94 (n=10)
– AVS	0.7 (n=10)	5.8 (n=10)	0.99 (n=10)	0.9 (n=10)	7.3 (n=10)	0.98 (n=10)
– HTX	0.4 (n=10)	3.4 (n=10)	1.0 (n=10)	1.9 (n=10)	16.1 (n=10)	0.84 (n=10)
**GRS**						
Overall	4.5 (n=50)	12.8 (n=50)	0.98 (n=50)	4.7 (n=50)	20.2 (n=50)	0.84 (n=50)
– Healthy subjects	5.7 (n=10)	13.0 (n=10)	0.86 (n=10)	5.0 (n=10)	15.4 (n=10)	0.42 (n=10)
– HF	3.0 (n=10)	15.7 (n=10)	0.85 (n=10)	4.6 (n=10)	17.7 (n=10)	0.51 (n=10)
– Perimyocarditis	4.2 (n=10)	13.3 (n=10)	0.97 (n=10)	3.7 (n=10)	15.6 (n=10)	0.77 (n=10)
– AVS	4.4 (n=10)	9.4 (n=10)	0.99 (n=10)	5.5 (n=10)	19.9 (n=10)	0.88 (n=10)
– HTX	6.1 (n=10)	14.7 (n=10)	0.96 (n=10)	5.3 (n=10)	20.3 (n=10)	0.77 (n=10)

**GCS**						
Overall	1.3 (n=50)	6.8 (n=50)	0.99 (n=50)	2.2 (n=50)	13.1 (n=50)	0.94 (n=50)
– Healthy subjects	1.6 (n=10)	7.2 (n=10)	0.89 (n=10)	1.8 (n=10)	9.0 (n=10)	0.70 (n=10)
– HF	1.0 (n=10)	7.7 (n=10)	0.96 (n=10)	1.6 (n=10)	7.9 (n=10)	0.69 (n=10)
– Perimyocarditis	0.6 (n=10)	3.5 (n=10)	1.0 (n=10)	2.5 (n=10)	18.3 (n=10)	0.88 (n=10)
– AVS	0.9 (n=10)	4.4 (n=10)	1.0 (n=10)	1.7 (n=10)	10.3 (n=10)	0.98 (n=10)
– HTX	1.9 (n=10)	9.1 (n=10)	0.97 (n=10)	3.0 (n=10)	13.3 (n=10)	0.93 (n=10)

Data are absolute numbers.

FT-CMR, Feature tracking cardiac magnetic resonance; SEE, Standard error of estimate; CV, coefficient of variation; ICC, Intraclass correlation coefficient; GLS, Global longitudinal strain; GRS, Global radial strain; GCS, Global circumferential strain; HF, heart failure; AVS, Aortic valve stenosis; HTX, heart transplantation.

Supplementary table 5 – Intra-observer and inter-observer reproducibility for myocardial strain evaluation using STE among participant subgroups

	Intra-observer			Inter-observer		
	SEE	CV (%)	ICC	SEE	CV (%)	ICC
**GLS**						
Overall	0.6 (n=50)	4.2 (n=50)	0.99 (n=50)	1.5 (n=50)	9.5 (n=50)	0.97 (n=50)
– Healthy subjects	0.6 (n=10)	3.3 (n=10)	0.97 (n=10)	1.5 (n=10)	8.1 (n=10)	0.79 (n=10)
– HF	0.7 (n=10)	5.5 (n=10)	0.97 (n=10)	1.0 (n=10)	9.0 (n=10)	0.89 (n=10)
– Perimyocarditis	0.5 (n=10)	2.9 (n=10)	1.0 (n=10)	1.2 (n=10)	7.8 (n=10)	0.98 (n=10)
– AVS	0.5 (n=10)	4.0 (n=10)	1.0 (n=10)	1.7 (n=10)	11.3 (n=10)	0.97 (n=10)
– HTX	0.9 (n=10)	5.8 (n=10)	0.99 (n=10)	1.3 (n=10)	9.0 (n=10)	0.95 (n=10)
**GRS**						
Overall	4.2 (n=39)	15.1 (n=39)	0.96 (n=39)	8.1 (n=33)	26.7 (n=33)	0.88 (n=33)
– Healthy subjects	5.7 (n=7)	16.6 (n=7)	0.94 (n=7)	9.4 (n=6)	24.2 (n=6)	0.81 (n=6)
– HF	1.8 (n=8)	12.6 (n=8)	0.97 (n=8)	4.1 (n=8)	22.5 (n=8)	0.90 (n=8)
– Perimyocarditis	4.7 (n=7)	14.9 (n=7)	0.91 (n=7)	13.5 (n=6)	38.9 (n=6)	0.75 (n=6)
– AVS	5.5 (n=8)	19.7 (n=8)	0.87 (n=8)	6.9 (n=8)	21.9 (n=8)	0.77 (n=8)
– HTX	2.6 (n=9)	7.8 (n=9)	0.99 (n=9)	4.5 (n=5)	21.0 (n=5)	0.83 (n=5)

**GCS**						
Overall	0.9 (n=39)	6.3 (n=39)	0.99 (n=39)	2.2 (n=33)	16.9 (n=33)	0.93 (n=33)
– Healthy subjects	1.7 (n=7)	8.7 (n=7)	0.84 (n=7)	1.7 (n=6)	8.6 (n=6)	0.75 (n=6)
– HF	0.7 (n=8)	7.1 (n=8)	0.97 (n=8)	2.2 (n=8)	23.1 (n=8)	0.69 (n=8)
– Perimyocarditis	0.9 (n=7)	5.8 (n=7)	0.99 (n=7)	1.7 (n=6)	18.8 (n=6)	0.91 (n=6)
– AVS	0.5 (n=8)	3.0 (n=8)	1.0 (n=8)	2.4 (n=8)	17.3 (n=8)	0.94 (n=8)
– HTX	0.8 (n=9)	5.3 (n=9)	0.99 (n=9)	2.3 (n=5)	19.9 (n=5)	0.38 (n=5)

Data are absolute numbers.

STE, speckle tracking echocardiography; SEE, Standard error of estimate; CV, coefficient of variation; ICC, Intraclass correlation coefficient; GLS, Global longitudinal strain; GRS, Global radial strain; GCS, Global circumferential strain; HF, heart failure; AVS, Aortic valve stenosis; HTX, heart transplantation.

Supplementary table 6 – Test-retest reliability for myocardial strain evaluation using FT-CMR and STE

	SEE	CV (%)	ICC
**FT-CMR**			
Healthy subjects			
– GLS	0.9 (n=10)	5.6 (n=10)	0.88 (n=10)
– GRS	10.6 (n=10)	28.5 (n=10)	0.16 (n=10)
– GCS	2.8 (n=10)	14.6 (n=10)	0.44 (n=10)
**STE**			
Healthy subjects			
– GLS	0.8 (n=10)	5.0 (n=10)	0.87 (n=10)
– GRS	6.0 (n=5)	21.9 (n=5)	0.88 (n=5)
– GCS	3.0 (n=5)	16.7 (n=5)	0.38 (n=5)

Data are absolute numbers.

FT-CMR, Feature tracking cardiac magnetic resonance; STE, speckle tracking echocardiography; SEE, Standard error of estimate; CV, coefficient of variation; ICC, Intraclass correlation coefficient; GLS, Global longitudinal strain; GRS, Global radial strain; GCS, Global circumferential strain.
